# Autonomous Image-Based Corrosion Detection in Steel Structures Using Deep Learning

**DOI:** 10.3390/s24113630

**Published:** 2024-06-04

**Authors:** Amrita Das, Sattar Dorafshan, Naima Kaabouch

**Affiliations:** 1 Department of Civil Engineering, College of Engineering & Mines, University of North Dakota, Grand Forks, ND 58202, USA; sattar.dorafshan@und.edu; 2 Department of Electrical Engineering, School of Electric Engineering & Computer Science, University North Dakota, Grand Forks, ND 58202, USA; naima.kaabouch@und.edu

**Keywords:** steel structure, corrosion, semantic segmentation, transfer learning, artificial intelligence

## Abstract

Steel structures are susceptible to corrosion due to their exposure to the environment. Currently used non-destructive techniques require inspector involvement. Inaccessibility of the defective part may lead to unnoticed corrosion, allowing the corrosion to propagate and cause catastrophic structural failure over time. Autonomous corrosion detection is essential for mitigating these problems. This study investigated the effect of the type of encoder–decoder neural network and the training strategy that works the best to automate the segmentation of corroded pixels in visual images. Models using pre-trained DesnseNet121 and EfficientNetB7 backbones yielded 96.78% and 98.5% average pixel-level accuracy, respectively. Deeper EffiecientNetB7 performed the worst, with only 33% true-positive values, which was 58% less than ResNet34 and the original UNet. ResNet 34 successfully classified the corroded pixels, with 2.98% false positives, whereas the original UNet predicted 8.24% of the non-corroded pixels as corroded when tested on a specific set of images exclusive to the investigated training dataset. Deep networks were found to be better for transfer learning than full training, and a smaller dataset could be one of the reasons for performance degradation. Both fully trained conventional UNet and ResNet34 models were tested on some external images of different steel structures with different colors and types of corrosion, with the ResNet 34 backbone outperforming conventional UNet.

## 1. Introduction

Corrosion is defined as changes in metal properties due to reactions with a surrounding corrosive environment, which deteriorates the metallic object’s functionality [1]. External or internal corrosion can reduce any steel structure’s lifespan [2].

Many types of defects occur in highway ancillary structures; however, corrosion is the most common [3]. Highway ancillary structures are exposed to weathering effects such as snow and rain, which lead to corrosion over time. The annual cost of corrosion damage estimated by the National Association for Corrosion Engineers (NACE) in 2016 was USD 2.5 trillion, which is equivalent to 3.4% of global GDP; however, current corrosion inspection techniques can save 15–35% of this estimated amount [4]. Among numerous corrosion monitoring techniques, ultrasonic testing is the most common one that can be used to get an instantaneous indication of cracks or corrosion. In this case, the traveling speed of the wave is generally considered for structural condition assessment. However, the authors of [5] reported a discrepancy in results in the case of multiple layers on a substrate. However, the performance of acoustic emission and eddy current technology in corrosion detection is not dependent on the number of layers and the size of defects [6]. These two methods may suffer from the recording of noisy signals and sensitivity to lift-off effects [6,7]. Regularly implemented inspection methods are not typically non-contact but non-destructive techniques (NDTs) that require a human inspector on-scene. Inspection accuracy depends on the inspector’s expertise and the accessibility of the defect location [8]. In addition, the road closures and traffic controls required for the maintenance and protection of traffic (MPT) could make inspections challenging to perform [9].

Many departments of transportation (DOTs) use unmanned aerial systems (UASs) for bridge inspections, allowing them to mitigate the problems related to manual inspections [10], which might be followed by the use of conventional image processing methods to detect steel corrosion [11,12,13,14]. The problems related to conventional image processing techniques are inaccurate user-defined parameters and adverse lighting condition effects [11]. The effect of over-pixelated regions in images may make conventional image-based methods less accurate [11]. Most researchers have used images collected in the lab to solve this problem, since the environment is controlled; however, this technique may produce a less generic defect detection model [12,13,14]. Using artificial intelligence (AI) as an alternative to the existing manned corrosion detection techniques can be a feasible solution to this problem.

Deep learning methods have already achieved a high degree of success in fields such as civil engineering, health care, cybersecurity intelligence, and smart cities [15]. This method’s intelligent performance and learning capacity from big data make it a key component in building smart data-driven systems [16]. Deep learning and machine learning are the two primary branches of artificial intelligence. The primary concept of artificial intelligence is the transfer of intelligence by duplicating the architecture of the human brain [17]. The primary difference between deep learning and machine learning is network depth and network architecture complexity [16]. Feature extraction in deep learning is also autonomous through the convolution layers. K-mean clustering, support vector machines, random forest, and decision tree are some user-defined shallow structures used for feature extraction in machine learning [18].

A machine learning algorithm may be overwhelmed by the exponential growth in data volume, unlike deep learning models [16]. The exponential development of new deep learning models could be overwhelming for structural defect detection, where annotated datasets are rare and expensive to create. Some already developed model architectures, such as Faster RCNN [19,20] and Mask RCNN [21], have been used by researchers to detect corrosion in the structural elements of steel bridges for this reason. Other deep convolution neural networks, such as ResNet50 [22,23], AlexNet [23], VGG-16 [23], and GoogleNet [23], have been used to detect metallic corrosion. AlexNet outperformed the other models in terms of correct corroded image predictions; however, the training dataset was imbalanced, and the proportion of damage in the test dataset was not reported. The authors of [24] developed a shallow convolutional neural network to predict corrosion in visual images using sliding windows of different sizes and scored a 98% recall value for a window size of 128 × 128 megapixels. Bolton et al. [25] tested five image classification models to detect corrosion in steel structures by initializing the random weights, including VGG16, ResNet50, AlexNet, Bastian Net, and ZF Net. VGG16 and ResNet50 were used in both transfer learning and fully trained modes. Two pre-trained models, VGG16 and ResNet 50, yielded a 98% recall value in detecting metallic corrosion in 900 images.

Researchers have come to a consensus on the suitability of models for different types of corrosion. For example, Jin et al. [19] suggested using Faster RCNN to detect corrosion; however, Rahman et al. [26] suggested using a supervised pre-trained semantic segmentation model, DeepLab, to detect corrosion at various levels instead of using an object detection model such as Faster RCNN, since a bounding box might not be suitable for corroded pixels with an irregular shape. The DeepLab model was tested on 100 images and detected corroded pixels with an accuracy of 72%; however, the model accurately predicted 93% of the non-corroded pixels. Forkan et al. [27] used ensemble networking with shallow networks, such as VGG16, to identify corrosion and Mask-RCNN to separate the region of interest. Ahuja et al. [28] established that residual UNet could efficiently detect pitting corrosion with a 98% recall value, which was higher than RCNN and SVM. Bastian et al. [29] used modified ZFNet to detect and localize different levels of corrosion in a pipeline with a 98.8% classification accuracy. Huang et al. [30] developed two lightweight models with residual blocks by modifying ShuffleNetV2 to segment metallic corrosion with a more than 85% mean IOU. Researchers have also fine-tuned some layers of pre-trained networks, such as ZFNet [31], PSPNet [32], DeepLab [32], and SegNet [32], to improve model performance.

Corrosion of industrial steel structures is also a serious matter to consider. Pre-trained PSPNet and Mask RCNN efficiently separated corroded pixels in a steel structure, with mean IOU values of 84.1% and 86.4%, respectively [33]. Katsamenis et al. [34] revealed that the Mask RCNN semantic segmentation model outperformed a fully convolution neural network and UNet, an encoder–decoder network, in terms of F1 score. Jiang et al. [35] used fusion attention UNet to detect corrosion in a steel box girder. Woo et al. [36] added a fusion module in place of the skip connection and a convolution block attention module (CBAM) to create a model they called modified UNet, which had a 77.75% mean IOU [35].

Many researchers [35,37] have compared UNet’s performance to that of existing convolutional neural networks, including AlexNet, Vgg16, and GoogleNet, to detect corrosion. The performance of modified UNet with the inclusion of a custom layer has also been investigated [35]. The purpose of this study is to evaluate the performance of UNet as a corrosion detection semantic segmentation model with different backbones under transfer learning and fully trained modes. The motivation of choosing a U-shaped network for this investigation is pixel-level accuracy in corrosion detection [35,37] and the reduced number of images required for training [36]. Moreover, transferring the contextual information to the expansion path enables the model to generate a precise segmentation map [37]. There are several deep learning libraries and resources [38], such as Pytorch [39] and TensorFlow [39]. The authors used Keras, a high-level neural network library built on the TensorFlow library, to train the semantic segmentation model.

## 2. Theoretical Concept of Artificial Neural Network

### 2.1. General Architecture of Deep Learning Models

A deep learning model’s skeleton is based on human neurons. The fundamental idea of a deep learning network unit is that an input (*x*) is weighted by *w* and bias (*b*), then summed. Here, bias (*b*) is a scalar value; however, the input (*x*) and weight (*w*) are vectors. The whole process can be described using Equation (1) [40]:

(1) $$y=\varphi \left(w^{T}x+b\right)$$

where φ is the activation function that executes non-linear transformation on the outputs from the convolution layers.

**Figure 1:**
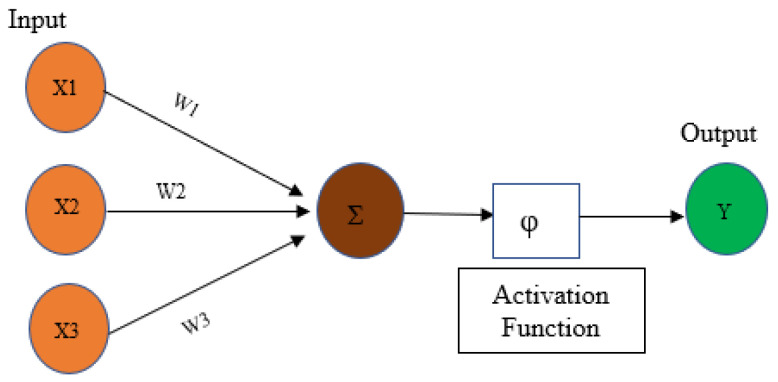
Simplified representation of a deep neural network architecture.

All neurons are generally interconnected with the neurons of the next layer. Each connection represents a parameter in the network. Local connectivity between the neurons reduces the number of parameters [40]. For example, the chosen kernel dimension is 3 × 3 × 1, and the number of filters is 1 for an input size of 10 × 10 × 1; therefore, the weight in a single filter sized 3 × 3 would be 9, bias = 1, and the total number of parameters from each filter is (9 + 1) or 10. A kernel is shared between the local neurons to collect the feature information. All accumulated information from the neurons is stored in a matrix, which is denoted as an activation map. The number of activation maps depends on the number of kernels, which are defined in the hyperparameters. The transferring of information is referred to as weight sharing in a convolutional neural network [41]. This working principle of convolution layers enables the network to handle large datasets. A simplified representation of a deep neural network structure is depicted in Figure 1.

### 2.2. Convolution

**Figure 2:**
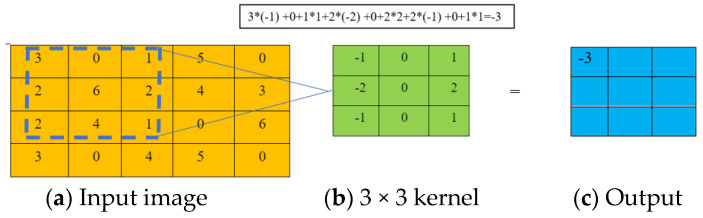
(**a**–**c**) Schematic presentation of convolutional operations.

Neural networks extract features from the input through the convolution layers by applying filters [41]. Filters are composed of differently sized kernels. Kernels operate in an area are of same size as the subarrays from the input data. Kernel weights can be initiated with random values and later updated as the training progresses. The weight values can be initiated randomly, or the pre-trained weights can be adopted in a pre-trained network. Kernels with specific sizes slide over the input image. The dot product is computed between each number of the kernel as the kernel moves, and the overlapped element of the input image is computed. The summation of the results from each multiplication identifies the output feature map (Figure 2a–c). This process is then repeated for the entire image. Kernel size is typically 3 × 3 but can be 5 × 5 or 7 × 7 [41].

### 2.3. Pooling Layer

Inserting a pooling layer between two convolutional layers plays a vital role in reducing the dimensionality of the output [42]. Pooling layers reduce the output dimension and the chance of overfitting [43]. The output image from one convolution layer is divided into small blocks during the pooling step, and a value from each block is selected to generate a compressed feature map. The two most used pooling methods are average pooling [44] and max pooling [45]. There may be one or more fully connected layers after the convolution and pooling layers [46,47,48]. Local features from the previous output are summed into global features by the fully connected neurons in these layers.

**Figure 3:**
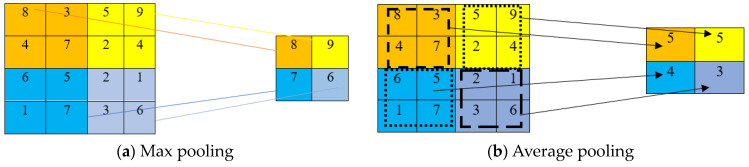
(**a**,**b**) Schematic presentation of different pooling operations.

A sliding window passes over the input and feeds the window number to a pooling function. A pooling function can be classified as max and mean pooling [49]. The maximum value from each extracted patch is stored as output and ignores all other smaller values in a max-pooling operation. Max and average pooling operations with a 2 × 2 filter and a stride of 2 are described in Figure 3a,b. Plane dimensions, such as height and width, are downsampled by a factor of two during the pooling operation.

### 2.4. ReLu Activation Function

Neural networks cannot manage complex features without an activation layer such as Tanh, Sigmoid, Leaky ReLu (Rectified Linear Unit), or ReLu. Among these, ReLu has become popular because of its simplicity. The main advantage of ReLu is that it does not activate all neurons together, unlike the other activation functions. Equation (2) states the functionality of ReLu such that if the value is less than 0 it is replaced with 0 [50], which means that the neurons become deactivated for negative output values.

(2) $$f\left(x\right)=\left\{\begin{array}{cc} 0, & x<0\\ x, & x>0 \end{array}\right.$$

### 2.5. Learning Mode of Deep Learning Models

Deep learning models can be trained in two learning modes, namely transfer learning and fully training mode. Full training mode refers to the stage when the models are trained from scratch; however, there are two types of approaches in transfer learning, namely (a) using a pre-trained model as a classifier and (b) including a fine-tuning step when the model is trained on the specific dataset [16]. Deep learning models typically need a large dataset compared to conventional machine learning models [18], which could limit their applications when these datasets are scarce or expensive to generate. It is also sometimes difficult to collect training data that have the same features as the test data [51]. Using a pre-trained model can solve this issue if the training dataset is relevant to the test set [52]. Transferring knowledge from an already trained model will save time and optimize the model’s learning.

Transfer learning has already been tested for steel defect detection [53]. The authors of [51] described transfer learning in two ways, namely as homogenous and heterogeneous. In the case of homogeneous transfer learning, the feature space of the source domain ($f_{s}$) is the same as the feature space of the target domain ($f_{t}$). Otherwise, this would be heterogeneous transfer learning. Transfer learning has its benefits; however, it could have adverse effects due to the irrelevance between the source and target domain [54]. This type of knowledge transfer is negative transfer learning. Negative transfer learning could be a result of class imbalance and the conditional difference between the source and target domains [55].

## 3. Methodology

This section first presents how the dataset was constructed. The learning efficiency of any machine learning model depends on the input dataset. Deep learning models have more layers than machine learning models, which help in improving the learning process. Despite this, an adequate number of representative training datasets still plays a significant role in artificial intelligence model performance, regardless of the number of layers [56,57]. Building up a network with a sufficiently large and representative dataset is challenging. Data augmentation can be a feasible solution for enabling a model to learn better [53]. The developed data augmentation scheme and metrics used for evaluation are reviewed below, and implementation details are provided.

### 3.1. Dataset Construction and Annotation

No datasets related to corrosion damage segmentation were available prior to this work; therefore, a new dataset was constructed from scratch. A cellphone camera (resolution: 13 megapixels) was used to capture 300 images of four in-service traffic poles with heights of 7 m between 10 a.m. and 12 p.m. in sunny conditions. The poles were in Grand Forks, North Dakota. These poles were later replaced due to the presence of severe corrosion. The image size was 2322 pixels by 4128 pixels. The images were collected for differing degrees of corroded and sound regions with different background scenes. Some representative images were selected for annotation after collection. The Python label-studio image labeler was used for annotation. All representative images were split into sections with dimensions of 227 by 227 pixels. A total of one hundred pieces with corroded pixels were used for annotation (Table 1). Only the corroded pixels were annotated; therefore, the method was semantic segmentation. Two other datasets were prepared with images collected by the authors using UAS and the Internet for qualitative model testing [58].

### 3.2. Data Augmentation

Some image augmentation libraries, such as imgaug [59], torchvision [39], Augmentor [60], CLoDSA [61], SOLT [62], and Automold [63], have been used over the last few years. The disadvantage of these libraries is the focus on the specific dataset/domain. Buslaev et al. [64] developed an augmentation tool named Albumentaion, which can provide versatility in image augmentation. The authors used the Python Albumentation library for data augmentation. Python LabelStudio in python 3.8 platform was used to semantically generate 100 binary masks from 100 representative images. The images and the masks were augmented to 2000 each, for a total of 2000 images and 2000 masks. Horizontal flip, vertical flip, translation, and grid distortion were used for augmentation (Table 2). The optimum number of augmentations per image was tested, and the optimum number range of the augmented images for each image was 20–40. The number of augmented images from one image was 20. Changes in the images were difficult to identify visually when the number of augmentations reached 40. 

### 3.3. Studied Model Architecture

In this study, UNet, which was developed for medical image segmentation [65], was used as a corroded pixel identifier with a different mode of training and pre-trained backbone. The details of different UNet architectures are discussed in the following sections.

#### 3.3.1. Encoder

**Figure 4:**
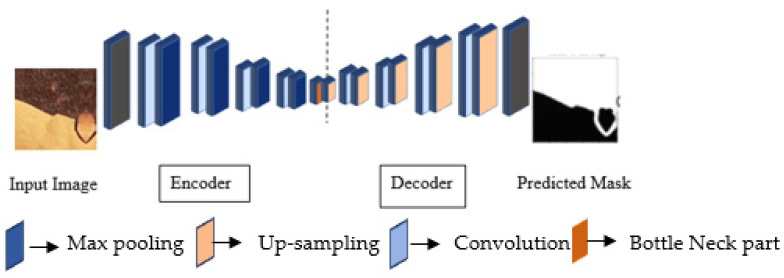
Schematic diagram of the U-shaped architecture of the encoder–decoder network.

The encoder consists of contraction blocks. The architecture of each block is similar to the conventional convolutional network (Figure 4). This study used three state-of-the-art networks, namely ResNet34, DenseNet121, and EfficientNetB7, as backbone feature extractors. The basic architecture of these convolutional neural networks is discussed below.

##### ResNet34

**Figure 5:**
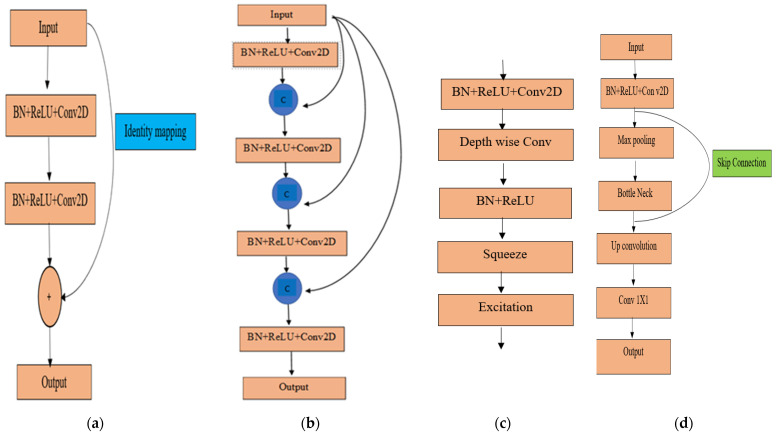
Unit block: (**a**) ResNet34, (**b**) DenseNet121, (**c**) EfficientNetB7, and (**d**) UNet.

ResNet 34 was introduced by He et al. [66]. The primary objective of this innovation was to reduce the difficulties in deeper neural network training. In a conventional convolution neural network, the output from the Nh layer is forwarded to the (N+1)th layer [67]; then, the output image is $X_{N}$ = *H_N_* ($X_{N}$ − 1). In the case of ResNet, a residual block was developed, which can be expressed using Equations (3) and (4).

(3) $$Y1=h{(X}_{N})+F{(X}_{N},W_{N})$$

(4) $$X_{N+1\;}=f(Y1)$$

where $X_{N}$ and $X_{N+1}$ are the input and output of the Nth unit, respectively; *F* is a residual function; and $W_{N}$ is the weight in the Nth layer. The term $h{(X}_{N})=X_{N}$ is identity mapping, and f is a ReLU [68]. This residual block helps in performing identity mapping without any computational complexities; therefore, it is termed a shortcut connection [67] (Figure 5a).

##### DenseNet121

DenseNet121 was developed by Huang et al. [67], with each layer connected to every other layer in a forward-feeding mechanism. DenseBlock (Figure 5b) is the primary building component of DenseNet121 [68]. Feature maps from the preceding layers are concatenated and passed to the subsequent layers with the current layer’s feature map to continue the feed-forward mechanism. For example, $X_{0}$, $X_{1}$, $X_{2}$……, and $X_{N}$ − 1 are the feature maps from all preceding layers of the Nth layer, and input of this layer can be expressed by Equation (5) [64].

(5) $$X_{N}=H_{N\;}(X_{0},\;X_{1},\;X_{2},\;\ldots \ldots ,\;X_{N-1})$$

where $H_{N}$ represents the Nth layer, $X_{N}$ is the output of the Nth layer, and $\left(X_{0},\;X_{1},\;X_{2},\;\ldots \ldots ,X_{N-1}\right)$ reflects the concatenation operation.

##### EfficientNetB7

EfficientNetB7 is a modified version of EfficientNetB0. A compound scaling method has been implemented on a baseline network, EfficientNet B0, to confirm the uniform scaling up of depth, width, and resolution, developing the advanced version known as EfficientNetB7 [69]. Mobile inverted bottleneck convolution (MBConv) is the basic building block of any EfficientNet architecture with squeeze and excitation optimization (Figure 5c) [70,71]. The output from the depth-wise convolution is forwarded to a new channel by pointwise convolution. Two layers of EfficientNet blocks are used for to squeeze and extend the channels [61]. Rectified Linear Unit (ReLU) and Batch Normalization (BN) are used to prevent information loss in the final layer of each block [72].

##### Original UNet

The structure of this network’s encoder block is the same as that mentioned in [65]. The input image passes through two 3 × 3 convolution layers and the activation function, ReLU. The down-sampling operation takes place with a max pooling operation with a stride of 2. The number of filters is doubled compared to the previous layer during down-sampling. This operation continues until reaching the bottleneck zone. The up-sampling of the feature map continues at the end of the bottleneck zone, which is the reverse of down-sampling. All feature maps from the corresponding layer are concatenated to avoid any loss of border pixels in the previous convolution layers. This concatenation operation is called a skip connection. The size of the final convolution layer is 1 × 1, confirming that the output size is the same as the input.

#### 3.3.2. Decoder

**Figure 6:**
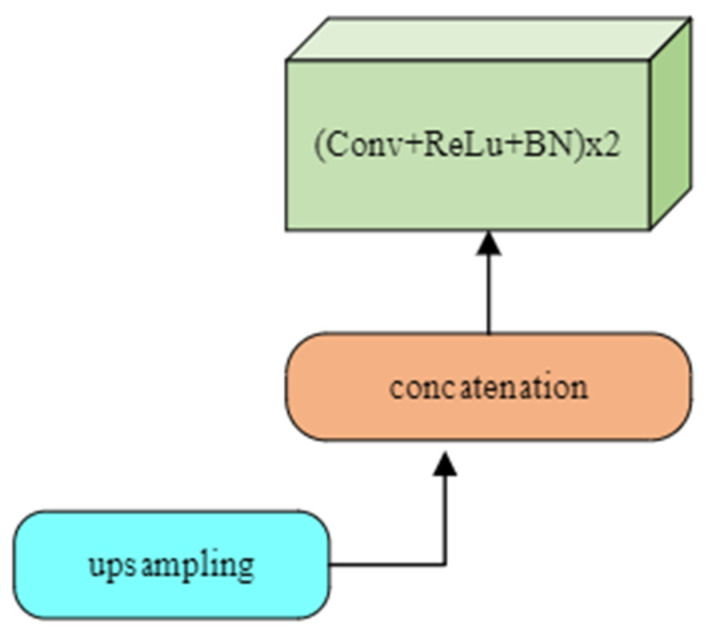
Unit block of decoder layers.

Up-sampling is performed in the decoder (Figure 6) to keep the size of the input and output images the same. Some features may be missing because of the use of max pooling during down-sampling. This problem is mitigated during up-sampling by using a filter size for each step that the same as the encoder. The output feature from each filter operation is copied and concatenated to construct the resultant segmented image of the same size as the input. This process is called transpose convolution. When up-sampling, all local information after filtering is integrated to obtain the full-resolution image. Each network’s decoder block (Figure 6) consists of sampling, concatenation, convolution, batch normalization, and activation operations. The number of filters reduces by half after concatenation, from 256 to 16, for all networks except original UNet. In the decoder part, the number of filters in UNet only decreases by half, from 256 to 64.

### 3.4. Model Training

**Figure 7:**
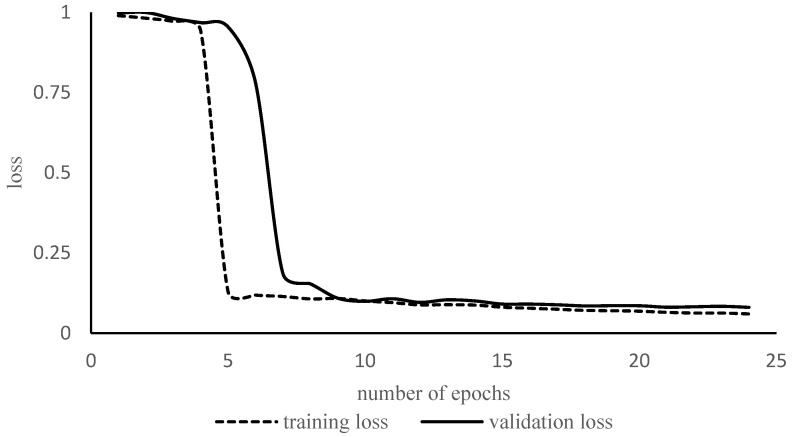
Training and validation loss curves of Resnet34 in full training mode.

All networks in transfer learning (TL) and fully trained (FT) mode were retrained/trained on the new dataset with 4000 images. Among the 2000 images and 2000 masks, 80% were used for training, 10% for validation, and 10% for testing. Augmentation was performed before training to confirm that the images with their respective mask should be picked by the model during training. The representative loss curve of Resnet34 is depicted in Figure 7. The model did not suffer from overfitting due to dataset augmentation. Figure 7 shows that there is a substantial gap between the training and validation loss curves until approximately 7–8 epochs. After epoch number 10, both the curves reached a point of stability. Hyperparameter selection was performed empirically after attaining knowledge from the literature review. The batch size is the number of images per iteration. The batch size was 16 images for each epoch. The initial learning rate is a parameter that determines the rate of learning. A higher learning rate means the network will learn faster, but the network might settle at a worse value. In this study, the learning rate was 0.001. The number of iterations is the number of times the network weights are updated. The number of epochs is the number of times the network has been updated for all the images in the training set. Here, the numbers of epochs and iterations for each model were 25 and 160, respectively. The number of parameters and training times are given in Table 3.

#### 3.4.1. Binary Cross-Entropy Loss Function

The binary cross-entropy loss function represents the performance map of a deep learning model that merges one or more variables into a real number [73,74]. There are several types of loss functions [75]. The most used loss function in machine learning is cross entropy [74], which is the difference between two probability distributions of a given set of variables. Selecting a loss function depends entirely on the type of segmentation and the number of classes. Binary cross entropy is used in a binary class model, and a categorical loss function can be used for a multiclass model. The target probability distribution (P) for the dataset would be 0 or 1 for our classification task, interpreted as sound and corroded pixels, respectively. Binary cross entropy can be defined using Equation (6).

(6) $$H\left(P,Q\right)=-\sum^{\left\{x\epsilon X\right\}}P\left(x\right)\times log(Q\left(x\right))$$

where each {xϵX} is a class label that is assigned to our dataset, P is the known probability of each class label, and Q is the probability predicted by the model. We established the P value as 0.5 or higher. The loss calculated by the binary cross entropy for a single image can be expressed using Equation (7).

(7) $$H\left(P,Q\right)=-(P(\mathrm{class}0)\times log(Q\left(class0\right))+(P(\mathrm{class}1)\times log(Q\left(class1\right))$$

#### 3.4.2. Optimizer

An optimizer’s goal is to minimize the loss function by updating the weights and biases in each iteration of training using the training rate. The most used optimizer is stochastic gradient descent (SGD); however, this optimizer needs to be tuned throughout the training process [76]. To mitigate this problem, the functionality of the learning rate has been modified according to different aspects and named Adagrad, Adadelta, RMS-Prop, and ADAM [45,77]. In this study, the ADAM optimizer was used to optimize the network. The working principle [78] of this optimizer can be described using Equations (8)–(11).

(8) $$\mu \left(t\right)=\beta_{1\;}\mu_{\left(t-1\right)\;}+(1-\beta_{1\;})g_{t}$$

(9) $$\gamma \left(t\right)=\beta_{2}\gamma_{\left(t-1\right)}-(\beta_{2}-1){\left[g_{t}\right]}^{2}$$

(10) $$∆w_{t}=-\frac{\alpha \times \mu \left(t\right)}{\sqrt{\gamma \left(t\right)}+\epsilon }g_{t}$$

(11) $$w_{t+1}=w_{t}+\nabla w_{t}$$

where $\mu \left(t\right)$ and $\gamma \left(t\right)$ are the first and second moment, of the gradients, respectively. $\beta_{1\;}$ and $\beta_{2}$ are the decay rate of the average of the gradients. The default values of $\beta_{1}$ and $\beta_{2}$ are 0.9 and 0.99, respectively, unless defined by the user [78]. α and ϵ are the learning rate and regularization term, respectively. $g_{t}$ is the gradient at time t. $w_{t}$ and $w_{t+1}$ are the weights at time t and t+1, respectively.

## 4. Results and Discussion

In this section, all results are presented and discussed in detail.

### 4.1. Evaluation Metrics

Semantic segmentation model performance evaluations should account for classification accuracy and localization correctness. In this study, model performance was evaluated using Equations (12)–(16).

(12) $$\mathrm{True}\;\mathrm{Positive}\;\mathrm{Rate}\;(\mathrm{TPR})=\frac{TP}{TP+FN}$$

where *TP* indicates the number of correctly detected pixels, and *FN* (false negative) indicates how many pixels were falsely detected as a negative class.

(13) $$\mathrm{True}\;\mathrm{Negative}\;\mathrm{Rate}\;(\mathrm{TNR})=\frac{TN}{TN+FP}$$

where *TN* indicates the number of correctly detected non-corroded pixels, and *FP* (false positive) indicates how many pixels were falsely detected as corroded.

This study evaluated the mean *IOU* or Jaccard Index for each model using Equation (14). Mean *IOU* is the metric by which the percentage of overlap between the ground-truth mask and the predicted mask is determined. This value ranges from 0 to 100%.

(14) $$\mathrm{Mean}\;\mathrm{IOU}\;\mathrm{or}\;\mathrm{Jaccard}\;\mathrm{Index}=\frac{TP}{TP+FP+FN}$$

*F*1 score measures the balance between the model’s precision and recall. *F*1 score is the metric obtained using Equation (15).

(15) $$F1\;\mathrm{score}=\frac{2TP}{2TP+FP+FN}$$

Pixel accuracy is the metric used to report the percentage of correctly classified pixels and can be determined using Equation (16).

(16) $$\mathrm{Accuracy}=\frac{TP+TN}{TP+FP+FN+TN}$$

### 4.2. Ablation Study

An ablation study investigates the effect of different layers of deep learning models by modifying the architecture or fine-tuning the network [78]. In this study, an ablation study was performed on UNet with different backbone variants, as mentioned below.

#### 4.2.1. Benefits of Knowledge from Transfer Learning

Two pre-trained convolution networks, DenseNet121 and EfficeintNetB7, were used as the backbone of the base model UNet. These two models were pre-trained on the ImageNet dataset. The encoder was kept frozen during training to evaluate the efficacy of the models’ pre-trained weights. All evaluation metrics indicated that EfficientNetB7 outperformed Densenet121 (Table 4); however, both models reached an average IOU of 90% or more, and the segmentation results were comparable in most testing images (Table 5). EfficientNetB7 had a 5% greater IOU than DenseNet121. The innovative and simultaneous compound scaling of network layers, width, and resolution results in a wider network with more feature storage [71]. As a result, EfficientNet reported approximately 4% fewer false-negative (FN) values. DenseNet was trained on 5 M of the parameters, corresponding to almost 50% of the total trainable parameters (12.2 M), which might have affected the memory in the retained weight from the pre-trained mode. Both models successfully detected non-corroded pixels, reporting TNR values above 95%. A detailed comparison between the DenseNet121 and EfficicentNetB7 predictions is presented in Table 5, establishing that 3823 pixels were annotated as corrosion in this specific test image. DenseNet121 predicted 4547 pixels as corrosion, out of which 724 were falsely predicted. EfficientNetB7 only predicted 420 pixels falsely, representing 9.8% of the total predictions.

#### 4.2.2. Effect of Training 

The model that performed the best in the previous section, EfficientNetB7, was selected to investigate the encoder–decoder model’s performance when trained from scratch. ResNet34 was chosen as a less deep network compared to EfficientNetB7. In addition to these models, a UNet with its original structure was built and trained from scratch. ResNet34 and UNet outperformed EfficientNetB7 in terms of all evaluation metrics (Table 6). The network performance of EfficientNetB7 dropped to a 35.5% TPR value when used in fully trained mode. EfficientNetB7 was proficient in detecting sound pixels, with a TNR value of 96.1%. It is difficult to obtain a standard dataset unless it is generated in a controlled environment; therefore, model performance may be affected when tested on images collected from the original structure that was exposed to the environmental conditions. The authors did not use any synthetic images to create a dataset with uniform corroded pixels to address this issue. Non-uniform defect sizes in terms of pixel value might deteriorate EfficientNetB7 performance. Srivastava et al. [75] established that deep networks with large parameters may learn complicated features; however, this property may lead the network to fit noise present in the training dataset when it is smaller, reducing the model’s ability to generalize. Our results validated the statement mentioned by Srivastava et al. [78] (Table 7). Compound scaling in EfficientNetB7 was designed so that the model should be trained on a high-resolution dataset [63]; however, the images were resized to 256 × 256 in this study. For this reason, comparatively shallow networks with fewer parameters to train (ResNet34 and UNet) can outperform the deeper EfficientNetB7.

ResNet 34 and UNet predicted approximately 94% of the corroded pixels correctly due to their shallows depth and less complex architectures. The IOU value ranged from 84 to 90%, which was higher than any semantic segmentation; however, UNet classified 4.5% more sound pixels as corroded, and ResNet34 classified 6.5% more corroded pixels as sound pixels. ResNet34 predicted the edges more sharply than UNet. The evaluation metrics mentioned in Table 6 present the average performance of the mentioned models, while Table 7 presents the performance of each model in a specific image. All models reported false predictions. ResNet34 and UNet yielded false predictions of 2.98% and 8.24%, respectively, whereas EfficientNetB7 detected 31% of the non-corroded pixels as corrosion.

### 4.3. Qualitative Comparison of ResNet34 and UNet

The model pre-trained on the external dataset and with different color components yielded less noisy segmentation than the fully trained model (Table 8). Both models performed satisfactorily when automatically detecting corroded pixels. The segmentation of the corroded pixels from the external images revealed the robustness of the ResNet34 and UNet models. Fully trained UNet did not segregate the background properly from the corroded part in one image, possibly due to the color similarity between the background and the corroded structure. We implemented UNet and ResNet34 as classifiers and trained them on 3200 images and masks for this test. Our dataset was small compared to big datasets such as ImageNet and CIFAR10 and consists of images from ancillary structures, which are yellow; however, the results established that the models were not biased to this color. The most important component of ResNet34 is the residual block, whereas it is the skip connection for the UNet architecture; therefore, a combination of both architectural features might help the model maintain a gradient, resulting in good predictions. All UNet and ResNet34 predictions are listed in Table 8.

In this study, all models were trained on the same dataset without confirming the optimized number of images necessary for each model. The demand for the number of images for the network with more depth, such as EfficientNetB7, may be higher than the number of images used for training. For this reason, an iterative training process should be performed by varying the number of images and learning rates to confirm the performance of the underperforming network, EfficientNetB7, in fully trained mode. This process is time-consuming and will be considered in future studies.

## 5. Conclusions

This study investigated the performance of a U-shaped encoder–decoder network for semantically segmented corroded pixels in steel structures with different backbones, along with the original structure. EfficientNetB7, ResNet34, and DenseNet121. A comparison was also made between the pre-trained and fully trained UNet for the autonomous segmentation of corroded pixels in steel structures. The models exhibited remarkable performance in different training modes. For example, EfficientNet reported a TPR of 97% and an IOU of 96.7% for the transfer learning mode; however, it reported a TPR of 35.53% TPR and an IOU of 35.43% for the fully trained mode. ResNet34 and the original UNet reported TPR and IOU values of approximately 90% and 85%, respectively. The results from the fully trained ResNet34 and original UNet models revealed that the shallower network performed well in corroded pixel determination in fully trained mode.

Another novel aspect of this study is the investigation of model performance when external datasets were used for testing. Two fully trained models were tested on the dataset that were independent in terms of dissimilarity with the training set and the type of sensor used during data collection. UNet with a ResNet34 backbone outperformed UNet with its original structure when using an external dataset. The most important component of ResNet34 is the residual block, which helps the model maintain gradient, resulting in better corroded pixel segmentation. The combination of a residual block with feature concatenation through skip connections helped the network collect and transfer important features from the preceding neuron.

The authors did not determine the number of optimum images necessary in fully trained mode, which could be a contributing factor when training deeper networks such as EfficientNetB7. An iterative training process should be performed by varying the number of images and learning rates to confirm the performance of the underperforming network, EfficientNetB7, in fully trained mode. This process is time-consuming and will be considered in future studies. A comparison of UNet with the other network architectures will also be included in further study.

## Figures and Tables

**Table 1:** Examples of data annotation.

Original Image	Annotated	Ground Truth (Binary)
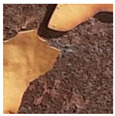	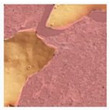	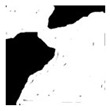

**Table 2:** Results obtained from different augmentation operations of the Albumentation library.

Original	Vertical Flip	Horizontal Flip	Random Rotate 90	Transpose	Grid Distortion
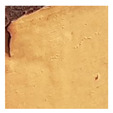	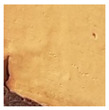	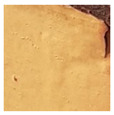	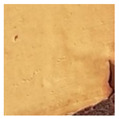	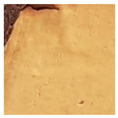	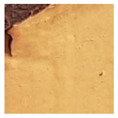
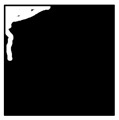	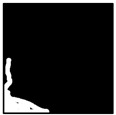	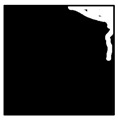	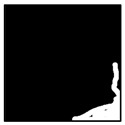	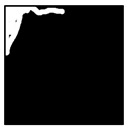	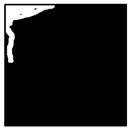

**Table 3:** Number of parameters for different models.

Backbone	Total Parameters (M)	Training Mode	Trainable Parameters (M)	Training Time (hrs)
DenseNet121	12.1	FT	12	6.8
		TL	5.2	5.82
EfficientNetB7	75	FT	74.7	16.35
		TL	11.2	15.43
ResNet34	24.4	FT	24	8.13
UNet	31.06	FT	31.04	14.4

**Table 4:** Evaluation metrics of UNet with different backbones in transfer learning mode.

Backbone	TPR (%)	TNR (%)	FPR (%)	FNR (%)	IOU (%)	F1 Score (%)	Accuracy (%)
DenseNet121	93.4	98.5	1.5	6.6	90.77	95.17	96.78
EfficientNetB7	97.3	99.1	0.9	2.7	95.6	97.8	98.5

**Table 5:** Comparison of predictions between the transfer learning modes of EfficientNetB7 and DenseNet121.

Original Image (Gray Scale)	Ground Truth	DenseNet121 Prediction	EfficientNetB7 Prediction
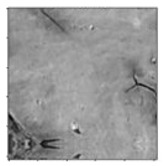	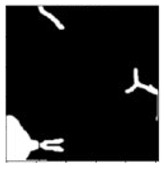	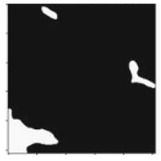	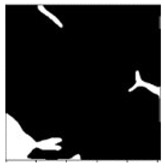
Number of corroded pixels	3823	4547	4243

**Table 6:** Evaluation metrics of UNet with different backbones in fully trained mode.

Base	TPR (%)	TNR (%)	FPR (%)	FNR (%)	IOU (%)	F1 Score (%)	Accuracy (%)
ResNet34	93.4	98.5	1.5	6.6	90.77	95.16	96.78
EfficientNetB7	35.5	96.1	3.9	64.5	35.43	52.32	38.68
UNet	93.9	94	6	0.1	84.11	91.4	94.02

**Table 7:** Comparison of predictions between full training modes of ResNet34 and UNet.

Original Image (Gray Scale)	Ground Truth	ResNet34 Prediction	UNet Prediction	EfficientNetB7 Prediction
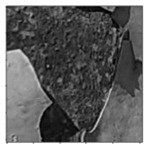	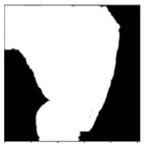	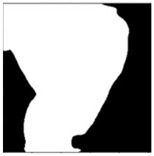	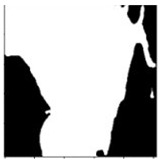	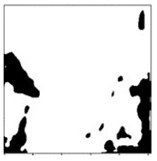
Number of corroded pixels	39,128	40,333	42,642	57,096

**Table 8:** Performance of fully trained UNet and ResNet34 on external images.

	Prediction by UNet	Prediction by ResNet34
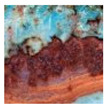	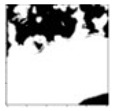	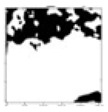
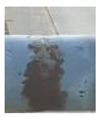	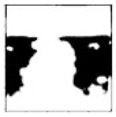	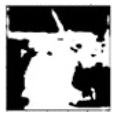
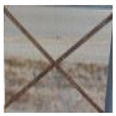	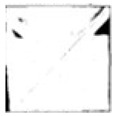	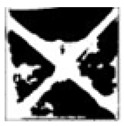
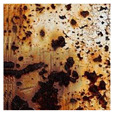	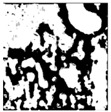	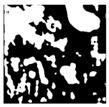

## Data Availability

Data are available upon request to the corresponding author.
